# Investigation of phenolic compounds and antioxidant properties in honey, pollen and propolis according to regional and *Apis mellifera* genotypes

**DOI:** 10.29219/fnr.v69.12234

**Published:** 2025-08-01

**Authors:** Semiramis Karlıdağ

**Affiliations:** Battalgazi Vocational School, University of Malatya Turgut Ozal, Malatya, Türkiye

**Keywords:** antioxidant, bee products, bees’ genotypes, flora, geographical location

## Abstract

**Objective:**

Honey, bee pollen and propolis are natural apicultural products whose chemical composition and biological activities are influenced by the floral characteristics of the regions in which they are produced. In this study, the phenolic profiles and antioxidant properties of honey, pollen and propolis samples collected from two distinct altitudinal zones (Yamadağ and Battalgazi) and derived from two different bee genotypes (*Apis mellifera caucasica* and *Apis mellifera carnica*) were systematically compared.

**Methods:**

The phenolic content of the ethanol extracts was quantified in terms of total phenolic content (TPC) and total flavonoid content (TFC). The phenolic composition was identified using HPLC-PDA (high-performance liquid chromatography – photodiode array detector), calibrated with 26 phenolic standards. The antioxidant activity was assessed using the ferric-reducing antioxidant power (FRAP) assay and diphenylpicrylhydrazyl radical scavenging activity.

**Results:**

The findings of this study demonstrate that both regional flora and bee genotype significantly affect the phenolic composition and antioxidant capacities of these bee products. Specifically, *A. m. carnica* was found to be more influential for pollen production, while *A. m. caucasica* exhibited greater significance in propolis. Furthermore, the results highlighted that, in terms of phenolic content and antioxidant potential, propolis exhibited approximately 300 times the richness of honey and 20 times the richness of pollen.

**Conclusion:**

Thus, the phenolic composition and antioxidant activity of bee products vary depending on factors such as regional flora, bee genotype and geographical location.

**Popular scientific summary**

- This study compared the phenolic composition and antioxidant activity of bee products obtained from different regions and bee genotypes.
- The results showed that factors such as regional flora, bee genotype and geographical location affect the phenolic compounds and antioxidant properties of honey, pollen and propolis.
- The antioxidant capacity of propolis was found to be stronger than honey and pollen.

Türkiye is in the northeastern hemisphere, serving as a bridge between the continents of Asia, Europe and Africa. It is surrounded by seas on three sides, with a unique position that contributes to its diverse climate. Due to its geographical location, proximity to seas and varied topography, Türkiye experiences a wide range of climate types, incorporating both Mediterranean and continental climate characteristics. With extensive coastlines along the Mediterranean and the Black Sea, Türkiye’s climate and ecosystems are highly diverse (1, 2).

The country boasts a rich and varied plant flora due to its distinct climatic zones, home to approximately 12,000 plant species, with around 30% of these being endemic to the region. This diversity in plant life also leads to a variety of honey-producing plants. Türkiye’s abundant and diverse flora supports a range of honey types, including floral honeys, honeydew honeys, forest honeys and those from industrial crops. Some of these honeys are monofloral, while others are heterofloral. While the majority of this honey is consumed domestically, it is also exported, particularly to European and Arab countries (3, 4).

Not only is the country rich in honey production but also in the biodiversity of bee products, such as honey, pollen and propolis, is Türkiye. The country’s diverse flora supports a wide variety of monofloral honey types. Some of the most commonly produced monofloral honeys include thyme (*Tilia spp.*)*,* heather (*Calluna vulgaris*), chestnut (*Castania sativa*), clover (Trifolium spp.), lavender (*Lavandula spp.*), sunflower (*Helianthus annuus*), lime (*Thymus spp.*), spiny restharrow, chaste tree (*Vitex spp*.), thistle (*Eryngium campestre*), acacia (*Robinia spp.),* black cumin (*Nigella sativa*), rhododendron, myosotis, etc. (5, 6). Additionally, the honeys are also produced from highland and plain regions (7–9). As for honeydew honey, pine honey (*Pinus spp*.) is the most prevalent, and the largest production of pine honey takes place along the Aegean and Mediterranean coasts of Türkiye (10). Other forest honeys, such as those derived from oak (*Querques spp.*), cedar (*Cedrus spp.),* willow (Salix spp.) and spruce trees (*Picea abies*), are also produced as honeydew honeys (11). These types of honey are harvested from the secretion of aphids and other insects that feed on tree sap, contributing to the unique characteristics of forest honeys (1, 3). In Türkiye, three main honeybee genotypes are commonly found: *Apis mellifera caucasica*, Apis *mellifera carnica* and *Apis mellifera anatoliaca*. *A. m. caucasica* is predominantly found in the Black Sea region and is known for its high adaptability to cold climates, resilience and productivity, particularly excelling in overwintering capacity and high honey yield. *A. m. carnica* is more commonly found in the Central Anatolia and Aegean regions, where it adapts well to warm climates and is known for its calm temperament and efficiency. This genotype is suitable not only for honey production but also for pollination and propolis production. *A. m. anatoliaca*, a native genotype specific to Türkiye, is primarily found in the Southeastern and Central Anatolia regions. This genotype is highly adapted to local conditions, exhibiting resilience, although its honey yield is generally lower compared to other species, it is highly successful in environmental adaptation (12, 13).

Honey, pollen, propolis, bee bread and royal jelly are significant bee products that, due to their nutritional and therapeutic benefits, have gained considerable recognition worldwide over the past quarter-century. They are particularly valued as food supplements and complementary medicine agents (14). Bee pollen is a substance collected by bees from flowers, containing the male reproductive cells of plants. Bees carry the pollen on their bodies and transport it back to their hives, where it is used as a food source (15). Bee pollen, which is also consumed by humans as a dietary supplement, is rich in vitamins, minerals, proteins and amino acids (5, 16). Propolis is a natural substance produced by bees using resin collected from plants. Bees use it to seal cracks in the hive, prevent microbial infections and maintain sterility. It is used by humans as a supplement to boost the immune system, with anti-inflammatory and antioxidant properties. Propolis is rich in flavonoids, phenolic acids and other bioactive compounds (17, 18).

The most abundant bioactive molecules in the bee products are polyphenols. The phenolic compounds are secondary metabolites of plants responsible for a wide range of biological activities, including antioxidants, antimicrobial, anti-inflammatory and antitumor properties (19, 20). The types and number of phenolic compounds in the bee products can vary depending on many factors such as the plant flora, seasonal conditions, the beekeeper’s performance and the geographical characteristics of the production area (2, 5).

In this study, we aimed to investigate the differences of honey, pollen and propolis according to different regions and bee genotypes in terms of apitherapeutically important secondary metabolites and antioxidant properties apart from their basic components (sugar, protein, minerals, etc.). The survey was conducted in two different geographical elevations within the Malatya region: Yamadağ and Battalgazi. The focus was on two *Apis mellifera* genotypes: carnica and caucasica. By analysing these variables, it was investigated how environmental factors and bee genetics influence the composition of bioactive substances in bee products.

## Materials and methods

### Samples

**Fig. 1:**
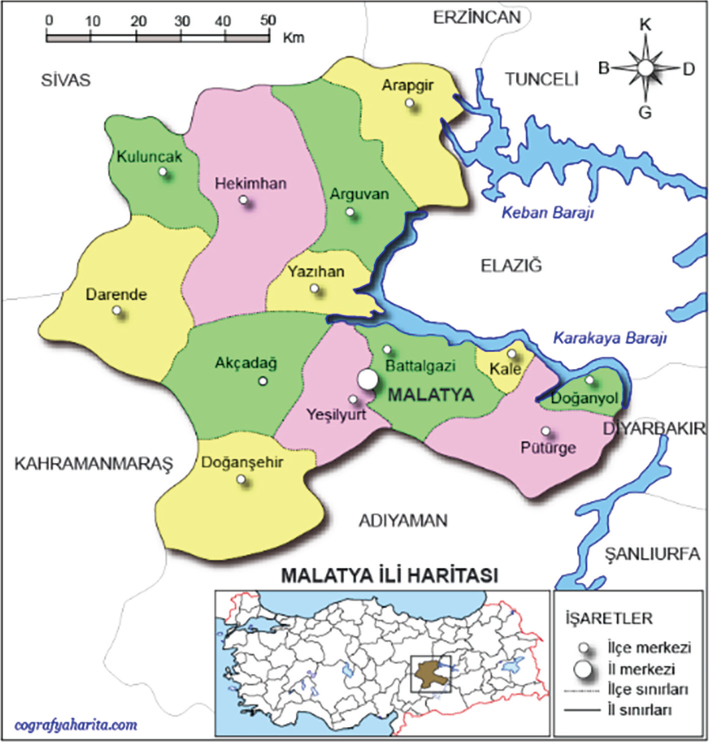
The research area is in Battalgazi and Yamadağ of Malatya.

This study was conducted in two different locations in Malatya province, located in the Eastern Anatolia Region of Turkiye. Detailed information about sampling is presented in Fig. 1 and Table 1 (21). The study was conducted in the apiary of Malatya Turgut Ozal University Battalgazi Campus Bee and Bee Products Development Application and Research Center. In the first week of April, a total of 80 colonies (40 Caucasian, 40 Carniolan) were equalised in terms of food amount and frame number. On June 7, 40 of these colonies were left in Battalgazi Campus and the other 40 were moved to Hekimhan/Yamadağ plateau. Harvesting continued until the second week of September.

**Table 1:** Data on honey, pollen and propolis samples

Sample name	Coordinates	Altitude (meter)
Yamadağ	38° 54′ 41″ N 38° 7′ 55″E	2,306
Battalgazi	38°25′22″N°38°21′56″E	885

Harvested honey frames were recorded with hive number, bee race and location information, filtered and stored in glass jars in a dark and dry environment for analysis. Pollen samples were collected from traps weekly between June 10 and September 10. Dry pollen samples, classified by region and bee genotype, were collected into 50 mL falcon tubes after collection and stored at +4 C for analysis. Propolis traps were placed in Battalgazi and Yamadağ colonies on June 9 and June 10, respectively, and were harvested on September 30 and stored at -18°C. They were mechanically separated from the traps and stored in glass jars before analysis.

### Palynological analyses of the honey

The botanical origin of the honey samples was determined by melissopalynological analysis. 10 mL distilled water was mixed with 10 g honey and heated at 50°C. It was then centrifuged at 3,500 rpm for 45 min. After centrifugation, the slide was prepared from the remaining residue with the glycerin gelatin matrix. Botanical characteristics were determined from pollen morphology using light microscopy (2, 3).

### Extractions of honey, bee pollen and propolis samples

A total of 10 g of honey was added to a 50 mL Falcon tube, followed by the addition of 30 mL of 70% ethanol. The mixture was subjected to ultrasonic bath treatment for 2 h and then vortexed for 24 h on a stirrer. After thorough filtration, the solution was centrifuged at 3,000 g for 10 min. The resulting supernatant was stored for further analysis (2). Five grams of powdered dry bee pollen sample were added to a 50 mL Falcon tube, followed by the addition of 30 mL of 70% ethanol. The mixture was first subjected to ultrasonic bath treatment for 2 h and then extracted for 24 h on a shaker. After filtration through a filter paper, the supernatant was stored at +4°C for further analysis (5). Three grams of powdered raw propolis were added to 30 mL of 70% ethanol, and the mixture was sonicated for 2 h. Subsequently, it was extracted for 24 h at room temperature using a shaker. After filtration, the supernatant was stored at +4°C for further analysis (22).

### Determination of total phenolic contents

The total phenolic content (TBC) was determined spectrophotometrically using the Folin-Ciocalteu method (23). For the analysis, the honey, pollen and propolis extracts were utilised by diluting them in different concentrations. For the analysis, 25 µL of the extract was combined with 400 µL of 0.2 N Folin-Ciocalteu reagent, diluted with 680 µL of distilled water and incubated at room temperature for 3 min. After incubation, 400 µL of 10% Na_2_CO_3_ solution was added, and the mixture was kept at 25°C for 2 h. The absorbance was measured at 760 nm using a spectrophotometer (Thermo Scientific Evolution TM 201, UV-VIS Spectrophotometer, Madison, USA). A calibration curve was constructed using six different concentrations of gallic acid standards ranging from 0.031 to 0.50 mg/mL. The TBC was expressed as mg gallic acid equivalents based on the standard curve.

### Determination of total flavonoid contents

The total flavonoid content (TFC) was determined using spectrophotometric assay (24). Initially, 25 µL of the ethanolic extract was mixed with 50 µL of 10% Al(NO_3_)_3_ and 50 µL of 1.0 M NH_4_CH_3_COO. The mixture was then diluted to 3.0 mL with 99% ethanol and incubated at 25 ºC for 45 min. After incubation, the absorbance was measured at 415 nm. A calibration curve was created using six different concentrations of quercetin standards ranging from 0.031 to 0.50 mg QUE/mL. The flavonoid content was expressed as mg quercetin equivalent (QUE) per 100 g of extract based on the standard curve.

### Determination of total antioxidant activity (FRAP assay)

The total antioxidant activity of the ethanolic extract was assessed using the ferric-reducing antioxidant power (FRAP) assay (25). The freshly prepared FRAP reagent, consisting of ferric tripyridyl-triazine (Fe-III-TPTZ), FeCl_3_, acetate buffer in 40 mM HCl and 2.5 mL of 20 mM FeCl3.6H2O solution, was mixed in a test tube. Subsequently, 3 mL of the FRAP reagent was combined with 100 µL of the extract and incubated for 4 min at 37°C. The absorbance was measured at 595 nm. The standard calibration curve was generated using varying concentrations of FeSO_4_.7H_2_O (ranging from 1,000 to 31.25 µmol/mL). The results were expressed as µmol FeSO4.7H_2_O equivalents per 100 g of the sample.

### Determination of DPPH radical scavenging activity

The diphenylpicrylhydrazyl (DPPH) radical scavenging assay was measured by the spectrophotometric assay (26). For this assay, firstly 1.0 ml of 0.04 mg/mL DPPH radical solution and 1.0 ml of the extract was mixed. The mixture was kept in the dark for 45 min at 25°C, and the absorbance was read at 517 nm. To calculate the SC_50_ value, samples were prepared by making six different dilutions of the minimum ethanolic extract and treated with DPPH solution and absorbed at 517 nm. The amount of extract scavenging 50% of this radical was determined from the graph as SC_50_.

### Determination of phenolic compositions by HPLC-PDA

The phenolic profile of the mixture was analysed using high-performance liquid chromatography – photodiode array detector (HPLC-PDA) (Shimadzu Liquid Corporation LC 20AT) and a C18 column (250 mm × 4.6 mm, 5 µm; GL Sciences). The calibration curve was established using 26 phenolic standards. The mobile phase consisted of two components: (A) 2% acetic acid in water and (B) a mixture of acetonitrile and water (70:30). The sample/standard injection volume was 20 µL, the column temperature was set at 30°C and the flow rate was maintained at 1.0 mL/min (27).

The ethanolic extracts were first subjected to liquid-liquid extraction for phenolic enrichment prior to analysis by HPLC-PDA. A 10 mL portion of the extract was concentrated under reduced pressure at 40 °C using a rotary evaporator. The resulting residue was re-dissolved in 10 mL of distilled water, and the pH was adjusted to two with concentrated hydrochloric acid. Afterward, three consecutive extractions were performed using diethyl ether and ethyl acetate to collect the organic phases. The solvents were then evaporated, and the remaining residue was re-dissolved in 2 mL of methanol. The solution was filtered through a 0.45 μm RC membrane and analysed for phenolic compounds (22).

### Statistics

All experiments were carried out in triplicate, and the results are presented as the mean ± standard deviation. Statistical analysis was performed using SPSS software (version 20). To compare the TPC, TFC, FRAP and DPPH, across different species, a one-way ANOVA was conducted, followed by Duncan’s multiple range test. Pearson’s correlation coefficient was applied to determine relationships between the tests.

## Results and discussion

The findings obtained as a result of the palynological examination of the preparations prepared from the honey samples obtained from the Battalgazi and Yamadağ region colonies are summarised in Table 2. As a result of palynological analyses of honey samples, *Verbascum, Xanthium* spp., Brassicaceae, Caryophyllaceae, *Onobbrychis* spp., *Vicia,* Fabaceae and *Trifolium* pollen were found in the Battalgazi region. On the other hand, Lamiaceae, *Hypericum, Astragalus* spp., *Vicia, Epilobium, Onobrychis*, *Trifolium,* Fabaceae, Apiaceae and Rosaceae pollen were identified in the Yamadağ region samples. Besides this, the *Vicia, Trifolium,* Fabaceae and *Onobrychis* spp. was determined in both groups (Table 2).

**Table 2:** Some information about palynological results of honey samples

Location	Pollen species	Types	Name
Battalgazi	*Verbascum, Xanthium* spp., Brassicaceae, Caryophyllaceae, *Onobbrychis* spp., *Vicia,* Fabaceae, *Trifolium*	Multi-floral	Mixed
Yamadağ	Lamiaceae, *Hypericum, Astragalus* spp., *Vicia, Epilobium, Onobrychis* spp., *Trifolium,* Fabaceae, Apiaceae, Rosaceae	Multifloral	Mixed

According to the census results, the ratios of pollen were determined, and the rates were classified as dominant pollen (45% and more), secondary pollen (16–44%), minor pollen (3–15%) and trace pollen (3% and less) (28). The variability of the components in the two samples indicated that honey was collected by the honeybee from different plants depending on the geographical location.

In a study conducted on colonies fed with different industrial sugars (15), in Battalgazi district *Cistus, Astragalus*, Poaceae, *Verbascum and Echium* pollen grains were the most frequent taxa for glucose group. For sucrose group *Astragalus, Cistus, Verbascum, Plantago* and Poaceae were dominant taxa. *Cistus, Astragalus, Verbascum, Berberi’s* taxa were arranged according to the value for bee feed group. For control group, the most widely represented taxa were mainly ranged as *Astragalus, Cistus, Artemisia, Verbascum, Plantago and Echium*. In a study examining 20 honey samples obtained from local beekeepers in Malatya (Doganyol) city, Türkiye (28), it was determined that 10 of the honeys were monofloral (lowest 46.51% Berberidaceae, highest 78.95% Asteraceae). An amount of 5.62% of the pollens belonging to the honeys used in the study were determined as dominant pollen, 13.48% as secondary pollen, 33.71% as important minor pollen and 47.19% as minor pollen. The results obtained in my study are similar to the literature results. In this study, the phenolic content and antioxidant properties of honey, pollen and propolis samples produced by two different bee species from distinct geographical regions were compared. The bee species investigated were *A. m. caucasica* and *A. m. carnica*, which are the most commonly utilised species in the region. The honey, pollen and propolis production was carried out by both bee species within the same region, with the hives located approximately 5 km apart.

The Yamadağ region, characterised by its high altitude, is a plateau that serves as a significant area for blossom honey production, particularly during the months of June and July. This region is also a preferred location for migratory beekeepers from various parts of Türkiye. In contrast, the Battalgazi region is a low-altitude, flat plain area that is ideal for honey production, especially in early June. For each bee species, the samples were collected from at least three different hives, and the resulting analysis values are summarised in Table 3. One of the most significant bioactive components in the honey, pollen and propolis is phenolic compounds, with the TPC being a key parameter in assessing their biological activity. These compounds play a crucial role in the antioxidant properties and overall health benefits of these bee products. The TBC serves as an important indicator for understanding the potential therapeutic and biological effects of the bee products (5, 11).

**Table 3:** The phenolic contents’ and antioxidant capacities’ mean value of the samples

Locations	Genotypes	TPC (mg GAE/100g)	TFC (mg QUE/100g)	FRAP (µmolFeSO_4_/100 g)	DPPH (mg/mL)
		Mean ± Std	Mean ± Std	Mean ± Std	Mean ± Std
**HONEY**					
Yamadağ	G1	29.33 ± 2.08**a**	3.96 ± 0.15**a**	273.20 ± 9.27**a**	36.71 ± 0.91**a**
	G2	25.00 ± 2.00**b**	3.86 ± 0.24**a**	252.37 ± 10.30**a**	39.41 ± 2.12**a**
Battalgazi	G1	19.47 ± 1.36**c**	2.66 ± 0.14**b**	120.87 ± 15.55**b**	46.63 ± 3.84**b**
	G2	23.00 ± 2.65**bc**	3.38 ± 0.38**c**	200.33 ± 19.60**c**	40.73 ± 0.54**a**
**POLLEN**					
Yamadağ	G1	458.00 ± 25.71**a**	170.67 ± 16.20**a**	49.75 ± 3.08**a**	0.99 ± 0.08**a**
	G2	640.67 ± 71.84**b**	281.00 ± 26.66**b**	115.25 ± 5.54**b**	0.76 ± 0.04**b**
Battalgazi	G1	701.67 ± 10.02**b**	296.00 ± 17.58**b**	82.61 ± 7.70**c**	0.71 ± 0.07**b**
	G2	846.67 ± 58.97**c**	339.33 ± 20.53**c**	132.29 ± 9.44**d**	0.46 ± 0.08**c**
**PROPOLIS**					
Yamadağ	G1	78.45 ± 5.50**a**	34.67 ± 1.15**a**	1385.33 ± 72.51**a**	59.67 ± 5.86**a**
	G2	69.67 ± 7.02**a**	28.67 ± 3.06**b**	1247.67 ± 89.81**b**	84.33 ± 10.26**b**
Battalgazi	G1	161.00 ± 6.24**b**	43.33 ± 3.06**c**	1577.33 ± 66.94**c**	68.67 ± 3.06**a**
	G2	130.00 ± 5.20**c**	43.67 ± 4.04**c**	1414.67 ± 45.80**a**	70.33 ± 6.03**a**

Statistical evaluations were achieved for each honey, pollen and propolis sample and the mean of each location separately. The same letters in each column were not significantly different at *P* < 0.05 (one-way ANOVA test). G1: *Apis mellifera caucasica*; G2: *Apis mellifera carnica*.

The TPC of the honey samples was found to range from 19.47 to 29.33 mg GAE/100 g, with the honey samples from the Yamadağ region exhibiting a comparatively higher TPC value. Significant differences were observed in the TBC of the honey collected from hives containing *A.m. caucasica* and *A.m. carnica* in both regions. Specifically, the TPC was higher in *A. m. caucasica* honey from the Yamadağ region, whereas honey from *A. m. carnica* exhibited a higher TPC in the Battalgazi region.

It was determined that the total phenolic and flavonoid contents of the honeys obtained from both regions were characteristic of light-coloured flower honeys with qualities appropriate to the regional flora. As a matter of fact, it is reported that the dominant plant species in the region are Astragalus, thyme and lavender, and the honey produced is from the light-coloured flower honey class and differs from dark-coloured honeys (such as chestnut and black cumin) in terms of phenolic components (2, 3, 7). Many studies have reported that the TPC in light-coloured flower honeys typically ranges between 15 and 35 mg GAE/100 g (6, 7). In contrast, dark-coloured honeys, such as chestnut (29), black cumin (18) and oak honey (3), exhibit higher TPC values, ranging from 40 to 100 mg GAE/100 g. The TFC of the honeys was found to range from 2.66 to 3.96 mg QUE/100 g, with the highest TFC observed in the honeys from the Yamadağ region. No significant differences in flavonoid content were detected between the bee genotypes. The results showed a positive correlation between TPC and TFC (*R*^2^ = 0.722, correlation is significant at *P* ≤ 0.01) (Table 4).

**Table 4:** Pearson correlation matrix on the honey, pollen and propolis samples x 5 variables

Correlation levels		TPC	TFC	FRAP	DPPH
**HONEY**					
TPC	Pearson correlation Sig. (two tailed)	1	0.722** 0.008	0.801** 0.002	-0.843** 0.001
TFC	Pearson correlation Sig. (two tailed)		1	0.958** 0.000	-0.790** 0.004
FRAP	Pearson correlation Sig. (two tailed)			1	-0.816** 0.002
DPPH	Pearson correlation Sig. (two tailed)				1
**POLLEN**					
TPC	Pearson correlation Sig. (two tailed)	1	0.951** 0.000	0.804** 0.002	-0.955** 0.000
TFC	Pearson correlation Sig. (two tailed)		1	0.815** 0.001	-0.919** 0.000
FRAP	Pearson correlation Sig. (two tailed)			1	-0.808** 0.001
DPPH	Pearson correlation Sig. (two tailed)				1
**PROPOLIS**					
TPC	Pearson correlation Sig. (two tailed)	1	0.869** 0.000	0.811** 0.001	-0.232 0.468
TFC	Pearson correlation Sig. (two tailed)		1	0.797** 0.002	-0.408 0.187
FRAP	Pearson correlation Sig. (two tailed)			1	-0.479 0.115
DPPH	Pearson correlation Sig. (two tailed)				1

TPC: total phenolic content; TFC: total flavonoid content; FRAP: ferric-reducing antioxidant power; DPPH: diphenylpicrylhydrazyl.

** Significant at 1% probability (*P* < 0.01).

** Correlation is significant at *P* ≤ 0.01) (Table 4).

Flavonoids are one of the largest and most biologically active subgroups within the polyphenol family, playing significant roles in plant physiology, such as pigmentation and pollinator attraction. They are commonly found in fruits, vegetables, tea and honey and are associated with various health benefits, including reducing the risk of cardiovascular diseases, improving cognitive function and offering protection against certain types of cancer (20, 30). Studies have reported that the TFC in honey is generally low, with values ranging from 4 to 8 mg GAE/g, particularly in dark-coloured honeys such as chestnut and pine honey (6, 10). The antioxidant capacities of the honeys were assessed using two methods. The FRAP method, a reliable technique for measuring total antioxidant capacity, indicates that a higher FRAP value corresponds to a stronger antioxidant capacity. The FRAP values of the honeys ranged from 120 to 273 µmolFeSO_4_/100 g, with honeys from the Yamadağ region exhibiting higher antioxidant capacity. Additionally, different results were obtained between the bee genotypes in both groups. The SC_50_ value, which indicates the DPPH radical scavenging activity, differs from the FRAP test in that a higher SC_50_ value reflects lower antioxidant activity. The lowest SC_50_ value was found in the *A. m. caucasica* honey from the Yamadağ region (Table 3). The results showed a strong negative correlation was determined between FRAP and DPPH (*R*^2^ = -0.816, *P* < 0.01 in Table 4). When comparing the FRAP and DPPH values of the honeys to those of different blossom honeys, it can be concluded that the blossom honeys from the Yamadağ region of Malatya possess higher antioxidant capacity than the blossom honeys from Kars and lavender honey from Isparta (2, 3).

In this study, the bee pollen samples were collected from two different regions and compared in terms of their phenolic components and antioxidant properties. The TBC of the pollen samples, composed of mixed flower pollen, was measured in mg GAE/100 g. The values ranged from 458.00 ± 25.71 to 846.67 ± 58.97 mg GAE/100 g, with the highest TPC found in the pollen samples from the Battalgazi region. Significant differences were observed in the TPC values of the pollens compared to the bee phenolics, with the pollen samples from *A. m. carnica* colonies exhibiting higher TPC values. While it was expected that the TPC values in pollen would vary based on regional flora, it was quite surprising to observe differences according to bee genotypes (Table 3).

The composition of bee pollen can be influenced by the bee genotype in various ways. Different genotypes may exhibit variations in foraging behaviour, plant preferences and pollen collection abilities, which can alter the type, quantity and diversity of pollen collected. Additionally, genotypes can impact the processing, storage and utilisation of pollen, affecting its chemical composition, phenolic content and antioxidant properties. Therefore, the bee genotype plays an important role in shaping the composition and quality of the pollen collected (31, 32). It was found that the TFC values in the bee pollen ranged from 171 to 339 mg QUE/100 g, and that the TFC values showed a parallel variation with the TPC values. The results showed a strong positive correlation between TPC and TFC (*R*^2^ = 0.951, *P* < 0.01) (Table 4). In a study comparing the phenolic content of various types of bee pollen, it was reported that the TPC values of all pollen types, except chestnut pollen, ranged from approximately 608–903 mg QUE/100 g. In a separate study, the TPC of chestnut pollen was reported to be around 3,000 mg QUE/100 g (5). In the same study, it was reported that the TFC values ranged from 153 to 420 mg QUE/100g, which is consistent with the findings of our study. It was found that the total antioxidant capacities of the pollen samples from the Battalgazi region were significantly higher than those from the Yamadağ region. Additionally, the pollen from *A. m. carnica* colonies in both regions exhibited higher antioxidant capacities in terms of both FRAP and DPPH activities. The results showed a strong negative correlation was determined between FRAP and DPPH (*R*^2^ = -0.808, *P* < 0.01 in Table 4).

The propolis samples obtained from beehives using traps were analysed in 70% ethanol extracts. In terms of total phenolic and flavonoid contents, it was found that the propolis samples from the Battalgazi region had a richer composition. Additionally, differences were observed between bee genotypes, with *A. m. caucasica* propolis exhibiting a higher phenolic content. The results showed a strong positive correlation between TPC and TFC (*R*^2^ = 0.869, *P* < 0.01) (Table 4). A moderate negative correlation was determined between FRAP and DPPH (*R*^2^ = -0.479, *P* < 0.01). It has been reported that propolis samples collected from different geographical regions of Anatolia exhibit a rich floral diversity, with the TBC in their ethanolic extracts ranging from 64 to 230 mg QUE/g (18). In the same study, it was shown that the TFC values ranged from 16 to 59 mg QUE/g. Numerous studies have shown that polyphenols are the primary biomolecules responsible for the antioxidant capacities of honey, pollen and propolis. However, particularly in honey and pollen, antioxidant-rich vitamins such as ascorbic acid, beta-carotene and alpha-tocopherols also play a significant role (33, 34). Polyphenols in bee products are not only responsible for antioxidant activity but also contribute to antimicrobial and antiviral properties (35, 36). The findings of the study support that the plant flora of the Battalgazi plateau is more favourable for pollen and propolis production. Indeed, the tree population in the region is as important as the flora for propolis production. Honeybees primarily collect propolis from tree leaves and resins exuded from trees. As the tree population decreases with increasing altitude, it becomes evident that the Battalgazi region is more suitable for propolis production.

The phenolic compositions of the honey samples from the Yamadağ and Battalgazi regions were analysed using HPLC-PDA with reference to 26 phenolic standards (Table 5). The quantities of phenolic compounds in the honey samples were calculated and expressed in µg/100g. It was determined that the majority of phenolic components in the honey samples from both regions were below the detection limits. Among the phenolic acids, *p*-coumaric acid and *t*-cinnamic acid were identified within detectable limits in the honey samples from both regions. Among the flavonoids, chrysin, pinocembrin, galangin and apigenin were found in significant amounts in the honey samples from both regions. However, hesperetin was exclusively detected in the honey samples from the Battalgazi region. No significant differences were observed between the regions or bee genotypes in terms of phenolic composition. Table 5 presents the presence and quantitative values of phenolic compounds in bee pollen samples from both regions, expressed in mg per 100 g. *p*-Coumaric acid, ferulic acid, *t*-cinnamic acid and caffeic acid were detected in the pollen samples from both regions. Caffeic acid, an important component of propolis, was found in higher concentrations in the pollen samples from the Yamadağ region. Ferulic acid and caffeic acid were found to be present in higher concentrations in the pollen samples in the *A.m. carnica* genotypes than the *A.m. caucasica.*

**Table 5:** Phenolic composition of honey, pollen and propolis samples using HPLC-PDA

Compounds	HONEY (µg/100 g)				POLLEN (mg/100 g)				PROPOLIS (mg/g)			
	Yamadağ		Battalgazi		Yamadağ		Battalgazi		Yamadağ		Battalgazi	
	G1	G2	G1	G2	G1	G2	G1	G2	G1	G2	G1	G2
**Phenolic Acids**												
Gallic acid	-	-	-	-	-	-	-	-	-	-	-	-
Protocatechuic acid	-	-	-	-	-	-	-	-	-	-	-	-
Clorogenic acid	-	-	-	-	-	-	-	-	-	-	-	-
p-OH Benzoic acid	**-**	**-**	-	-	**-**	**-**	-	-	**-**	**-**	-	-
Syringic acid	-	-	-	-	-	-	-	-	-	-	-	-
Vanillic acid	-	-	-	-	-	-	-	-	-	-	-	-
*p*-Coumaric acid	8	12	22	260	5.4	4.8	2.60	70,30	-	-	16.72	3.76
Ferulic acid	-	-	-	-	1.74	5.8	12.2	139.19	-	-	136.86	-
Cinnamic acid	10	32	8	4	-	-	-	-	4.35	3.48	-	3.34
Caffeic acid	-	-	-	-	2.79	2.87	48.03	144.40	17.94	-	45.45	16.41
Ellagic acid	-	-	-	-	13.71	18.26	-	-	1.56	1.97	6.68	1.58
**Flavonoids**												
Catechin	-	-	-	-	-	-	-	-	-	-	-	-
Epicatechin	-	-	-	-	-		-	-	-	-	-	-
Rutin	-	-	-	-	-	-	-	-	-	-	-	-
Myricetin	-	-	-	-	-	--	-	-	-	-	-	-
Resveratrol	-	-	-	-	-		-	-	-	-	-	-
Daidzein	-	-	-	-	-	-	-	-	-	-	-	-
Luteolin	-	-	-	-	-	-	-	-	-	-	-	-
Quercetin	-	-	-	-	-		-	-	3.88	8.92	-	6.22
Apigenin	1500	146	84	120	-		-	54.86	-	8.84	89.32	11.16
Hesperetin	-	-	54	228	-	-	-	-	18.90	6.07	124.01	20.39
Rhamnetin	-	-	-	-	-	-	-	-	10.18	16.01	100.40	17.16
Chyrisin	392	346	180	420	12.19	14.13	115.90	375.30	59.36	60.15	440.09	94.56
Pinocembrin	408	335	202	320	12.83	14.71	88.14	455.02	82.80	67.03	450.09	99.63
Galangin	407	353	202	370	12.23	15.50	109.50	580.98	83.28	99.12	110.20	82.68
CAPE	-	-	-	-	9.13	7.18	53.43	195.22	23.52	26.82	190.96	418.93

(-): not detected. G1: *Apis mellifera caucasica*; G2: *Apis mellifera carnica*. CAPE: caffeic acid phenethyl ester.

The phenolic compositions of honey, pollen and propolis samples were found to correlate with their TBC and TFC levels. Among the three, propolis exhibited the highest phenolic content, followed by pollen, with honey showing the lowest levels. The findings reveal regional differences in phenolic richness. It was determined that honey samples from Yamadağ region and pollen and propolis samples from Battalgazi region were richer in terms of phenolic content. Additionally, propolis samples from the *Apis mellifera caucasica* contained higher phenolic levels compared to those from the *Apis mellifera carnica*. The high phenolic content and antioxidant activity in the pollen and propolis from Battalgazi indicate that the region has richer floral resources. The region is abundant in fruit trees, including apricot, cherry, plum and peach, which contribute to its suitability for pollen and propolis production. In contrast, the Yamadağ region, dominated by alpine flowers, produces honey with higher antioxidant properties. However, the high-altitude environment of Yamadağ lacks the tree cover necessary for propolis production, underscoring the critical role of tree availability in determining propolis yield (37). The Malatya region is globally renowned for its apricot production, quality and diversity, earning recognition under the name ‘Malatya apricot’ (38). In a previous study, the palynological properties of propolis from the Malatya region were analysed, revealing the presence of Poaceae (28.2%), Carduus (7.7%) and Astragalus (5.9%) species in the Battalgazi district (37). The region of the propolis samples exhibit higher levels of caffeic acid and caffeic acid phenethyl ester (CAPE) compared to propolis from many other regions of Anatolia (18, 39).

In conclusion, this study revealed that phenolic compositions and antioxidant properties of honey, pollen and propolis samples collected simultaneously from two regions of Malatya province with different altitudes, and floral characteristics were closely related to the regional flora. It was also found that bee genotypes affect honey, pollen and propolis collection behaviour. According to the samples from this region, the antioxidant capacity of propolis was found to be approximately 300 times higher than honey and 20 times higher than pollen.
